# Safety Monitoring of High-Risk Antibiotics Using Artificial Intelligence: A Narrative Review with Focus on Real-World Evidence

**DOI:** 10.3390/life16071158

**Published:** 2026-07-13

**Authors:** Mila Kostić, Marta Krpan, Paula Bulić, Martin Bobek, Jakov Kožić, Robert Likić

**Affiliations:** 1 School of Medicine, University of Zagreb, Zagreb 10000, Croatia; mila.kostic03@gmail.com (M.K.); krpanmarta@gmail.com (M.K.); paulabulic@gmail.com (P.B.); m.bobek77@gmail.com (M.B.); kozicjakov@gmail.com (J.K.); 2 Division of Clinical Pharmacology, Department of Internal Medicine, University Hospital Centre Zagreb, Zagreb 10000, Croatia

**Keywords:** artificial intelligence, drug safety, antimicrobial stewardship, pharmacovigilance, real-world evidence, high-risk antibiotics, therapeutic drug monitoring, outpatient parenteral antimicrobial therapy

## Abstract

High-risk antibiotics remain indispensable in contemporary infectious diseases practice, yet they account for a disproportionate share of preventable toxicity, therapeutic drug monitoring complexity, and antimicrobial stewardship workload. Vancomycin, aminoglycosides, colistin, linezolid, daptomycin, selected beta-lactams, and amphotericin B are particularly challenging because clinically relevant exposure-toxicity relationships coexist with marked inter-patient variability and fragmented post-marketing safety surveillance. Artificial intelligence and real-world evidence are increasingly proposed as complementary approaches to address these limitations, although the evidence base remains heterogeneous and predominantly retrospective. This narrative review synthesises literature from PubMed/MEDLINE, Scopus, and Web of Science published between 2019 and April 2026, supplemented by citation chaining and regulatory pharmacovigilance resources, with studies prioritised by implementation maturity, external validation status, and stewardship relevance. Current evidence indicates that artificial intelligence may improve safety monitoring when embedded within clinically rich data environments: machine learning models show promising discrimination for nephrotoxicity and haematological toxicity in vancomycin, colistin, and linezolid therapy; natural language processing may enhance adverse drug event extraction from clinical text; and Bayesian, model-informed tools already demonstrate clinical utility in vancomycin and aminoglycoside dosing. However, prospective implementation data remain sparse, external validation is uncommon, and evidence that these tools improve real-world antibiotic safety outcomes, as opposed to predictive discrimination alone, remains limited. Artificial intelligence-enabled antibiotic safety monitoring is therefore transitioning from methodological promise towards conditional clinical utility rather than proven benefit. Near-term value is most likely to arise from integration with therapeutic drug monitoring, antimicrobial stewardship, and pharmacology-led clinical review rather than autonomous decision-making, with clinical pharmacologists and stewardship teams leading local implementation, validation, and governance of these tools.

## 1. Introduction

High-risk antibiotics are agents for which the margin between therapeutic exposure and clinically important toxicity is relatively narrow, or for which toxicity is frequent enough to require structured monitoring, dose adjustment, and stewardship oversight. In routine hospital practice, this group includes vancomycin, aminoglycosides, colistin, linezolid, daptomycin, and selected beta-lactams such as cefepime, which is prone to concentration-related neurotoxicity in patients with renal dysfunction. Amphotericin B is the single exception to this otherwise antibacterial scope: it is antifungal rather than antibacterial, and it is included deliberately, not incidentally, because it shares with the antibiotics discussed here the same fundamental toxicity-monitoring problem of potent efficacy constrained by dose-limiting organ toxicity and dependence on laboratory surveillance. Its inclusion is therefore justified on toxicological and monitoring grounds rather than on antimicrobial class, and it is discussed throughout as a comparator rather than as a further antibacterial agent. These drugs are essential for multidrug-resistant infection, deep-seated Gram-positive disease, severe sepsis, and complex outpatient parenteral antimicrobial therapy (OPAT), yet they are also among the anti-infectives most likely to trigger nephrotoxicity, neurotoxicity, cytopenias, hepatotoxicity, or treatment interruption [1,2,3].

Antibiotic-related adverse drug reactions (ADRs) are common in hospitalised patients. Observational studies demonstrate that antibiotics are among the leading medication classes implicated in adverse events during admission, and meta-analytic evidence indicates that suspected ADRs are associated with prolonged hospitalisation, higher costs, and worse clinical outcomes in older inpatients [1,2,3]. The clinical burden extends beyond severe or irreversible toxicity. More frequent events, including rising serum creatinine during vancomycin therapy, linezolid-associated thrombocytopenia, or cefepime-associated encephalopathy, can force de-escalation, delay source control, prolong length of stay, and complicate stewardship decisions.

Traditional pharmacovigilance, while necessary, is insufficient for this challenge. Spontaneous reporting systems capture rare and unexpected safety signals but are constrained by under-reporting, duplicate submissions, missing exposure denominators, variable causality assessment, and delayed recognition of concentration-dependent toxicities [4,5]. These limitations are especially important for antibiotics because many toxic effects develop against a background of acute illness, polypharmacy, renal dysfunction, and rapidly changing physiology, making causal attribution difficult both at the bedside and in post-marketing surveillance. It is important to note that artificial intelligence does not resolve these fundamental limitations; it may, under certain conditions, attenuate some of them.

Artificial intelligence (AI) and real-world evidence (RWE) are emerging as practical responses to selected shortcomings of conventional pharmacovigilance. AI may expand the capacity to detect patterns within structured and unstructured data, while RWE broadens the evidence base beyond clinical trials by utilising electronic health records (EHRs), claims data, registries, spontaneous reporting systems, and digital monitoring tools. However, enthusiasm for these approaches must be balanced against the recognition that the field is currently experiencing an AI hype cycle that does not always translate into clinically validated tools. The central question is no longer whether AI can contribute to antibiotic safety monitoring, but whether and how specific applications can be rigorously validated, responsibly governed, and meaningfully embedded in clinical workflows [6,7,8,9].

The aim of this review is to summarise current evidence on AI-enabled safety monitoring of high-risk antibiotics, addressing the specific question of whether, and under what conditions, artificial intelligence and real-world evidence can be expected to improve detection, prediction, and prevention of antibiotic-related toxicity in routine clinical practice. We anticipated, and set out to examine against the available literature, that AI-enabled tools would show the most credible evidence of clinical utility in the narrowly defined domain of therapeutic drug monitoring and dose individualisation, and the least mature evidence where broader claims of autonomous safety surveillance are made. The review places emphasis on RWE sources, therapeutic drug monitoring (TDM), implementation barriers, and clinically relevant future directions for infectious diseases services, clinical pharmacologists, and antimicrobial stewardship (AMS) teams, and it deliberately prioritises critical appraisal of evidence quality over descriptive summaries of individual studies, framing AI primarily as a tool to support stewardship workflows rather than as an autonomous pharmacovigilance system. This synthesis is distinct from a recent narrative review addressing AI applications across bacterial infection management more broadly: rather than surveying diagnosis, antimicrobial selection, and infection management as a whole, the present review focuses specifically on safety monitoring, pharmacovigilance, TDM, and toxicity prediction for a defined set of high-risk agents, and applies a consistently critical lens to the distinction between predictive performance and demonstrated clinical benefit throughout.

## 2. Methods

This manuscript was designed as a narrative review rather than a systematic review. Its purpose was to provide an interpretive, clinically focused synthesis of an evolving literature spanning infectious diseases, clinical pharmacology, pharmacovigilance, biomedical informatics, and regulatory science. A formal protocol was not prospectively registered and no PRISMA flow diagram was prepared, as this is consistent with the narrative design. However, in recognition of the importance of methodological transparency, we describe the search and selection process in sufficient detail to allow critical appraisal.

### 2.1. Literature Search

A structured search was performed for publications indexed up to 30 April 2026 in PubMed/MEDLINE, Scopus, and Web of Science. The core PubMed search string combined terms for AI methods (artificial intelligence, machine learning, deep learning, natural language processing, Bayesian, large language models) with antibiotic-related terms (vancomycin, aminoglycosides, colistin, linezolid, daptomycin, beta-lactams, cefepime, amphotericin B) and pharmacovigilance-relevant terms (safety, toxicity, adverse drug reactions, pharmacovigilance, therapeutic drug monitoring, electronic health records, FAERS, EudraVigilance). The search strategy was adapted for Scopus and Web of Science using equivalent subject headings. Amphotericin B was included in the search string as the sole antifungal comparator because it shares the toxicity-monitoring and TDM problem that defines the antibacterial agents in scope; all other agents discussed in this review are antibacterial. Citation tracking and targeted hand-searching of regulatory documents, pharmacovigilance platform reports, and stewardship guidelines supplemented the database searches.

### 2.2. Study Selection and Prioritisation

The initial combined search identified an estimated 1200 records across databases after removal of duplicates. Consistent with a narrative rather than systematic methodology, titles and abstracts were read by two authors and discussed collaboratively, rather than screened through a formal independent dual-reviewer protocol, for relevance to AI-enabled antibiotic safety monitoring or TDM. Full-text review was applied to an estimated 180 records, of which 60 were selected as primary references on the basis of relevance and the prioritisation criteria described below, rather than through a formal eligibility algorithm. Differences in opinion were resolved by discussion and, where necessary, by involvement of a senior author. These figures are approximate and are reported for transparency rather than as evidence of a systematic methodology; the review is not intended to be exhaustive, no PRISMA flow diagram is presented, and its scope and prioritisation criteria are explicitly defined below.

We included English-language original studies, systematic reviews, meta-analyses, clinically informative narrative reviews, practice guidelines, and methodologically relevant papers. We prioritised, in approximate order of importance: (1) prospective or implementation studies demonstrating patient-level clinical impact; (2) externally validated models across multiple centres or institutions; (3) studies employing common data models enabling cross-institutional comparison; (4) stewardship-relevant workflows and governance frameworks; and (5) landmark methodological papers where no prospective equivalents existed. We excluded conference abstracts without substantive methods, purely in vitro or preclinical studies without clinical translation, and papers unrelated to antibiotic toxicity, pharmacovigilance, or RWE.

### 2.3. Acknowledgement of Evidence Limitations

The AI literature in pharmacovigilance is characterised by significant heterogeneity in study design, outcome definitions, AI methodology, validation strategy, and reporting standards. The large majority of published models are retrospective and internally validated at a single centre. Performance metrics are frequently reported as area under the receiver operating characteristic curve (AUROC) alone, without calibration data or decision-curve analysis. This review explicitly acknowledges these limitations and attempts to distinguish technical predictive performance from clinical utility and from actual implementation benefit throughout. Readers should interpret claims about AI capability in antibiotic safety monitoring with commensurate caution.

## 3. Current Evidence and Clinical Applications

### 3.1. High-Risk Antibiotics: Overview of Safety Concerns

The toxicological profile of high-risk antibiotics is dominated by predictable, exposure-related injury superimposed on unpredictable host factors. Nephrotoxicity remains the most extensively studied problem. For vancomycin, the transition from trough-based monitoring to area under the concentration-time curve to minimum inhibitory concentration (AUC/MIC)-guided monitoring reflects a consistent association between excessive cumulative exposure and acute kidney injury (AKI). Vancomycin nephrotoxicity is more likely in the presence of high daily doses, prolonged treatment, obesity, haemodynamic instability, and concomitant nephrotoxins, particularly in critically ill patients [10,11]. Importantly, confounding in these analyses is substantial: severity of illness, pre-existing renal dysfunction, and competing nephrotoxins all independently predict AKI, and their effects are not always separable from drug-attributable harm. This confounding challenge is one that AI models must explicitly address rather than ignore.

Aminoglycosides and colistin share the same operational challenge, though with different mechanistic emphases. Aminoglycosides cause dose-limiting nephrotoxicity and ototoxicity through renal cortical and cochlear accumulation, with risk determined by cumulative exposure, trough concentrations, and individual susceptibility [12]. Colistin-associated nephrotoxicity remains a dominant safety concern in treatment of multidrug-resistant Gram-negative infection, occurring in a substantial proportion of treated patients and often compounded by co-administration of other nephrotoxins in ICU settings [13]. Amphotericin B, while antifungal, illustrates the classic anti-infective problem of potent efficacy constrained by renal toxicity: it remains a useful comparator because it highlights the need for formulation-aware safety monitoring and the predictive value of cumulative exposure metrics [14].

Neurotoxicity is increasingly important for stewardship because it is frequently misclassified as sepsis-associated encephalopathy or non-specific neurological deterioration. Linezolid is associated with peripheral and optic neuropathy, serotonin toxicity, and lactic acidosis during prolonged therapy, while metronidazole can cause a characteristic yet under-recognised encephalopathy syndrome that is reversible if detected promptly [15,16]. Cefepime is the clearest example of a narrow therapeutic index beta-lactam with concentration-related neurotoxicity enriched by renal dysfunction, presenting with encephalopathy, myoclonus, aphasia, or non-convulsive status epilepticus [17]. The neurotoxicity associated with these agents is precisely the type of outcome where AI tools have potential clinical value, because EHR-derived laboratory trends, renal trajectories, cumulative exposure data, and clinician notes may collectively identify patients at risk before overt symptoms appear.

Cardiotoxicity is more heterogeneous across antibiotic classes. For macrolides, particularly azithromycin in susceptible patients, the concern centres on QT prolongation and arrhythmic risk, although population-level risk estimates are modest and residual confounding in observational data remains substantial [18]. Daptomycin is associated with creatine phosphokinase elevation, myopathy, and eosinophilic pulmonary syndromes rather than direct cardiotoxicity, but it remains operationally high-risk because these adverse effects require structured biochemical monitoring and may emerge during prolonged therapy or OPAT [19]. Haematological toxicity is particularly relevant for oxazolidinones: linezolid-associated thrombocytopenia typically develops after one to two weeks of treatment, occurs earlier in patients with renal impairment or low baseline platelet counts, and is now well enough characterised to support risk prediction and concentration-guided prevention strategies [15,20]. Hepatotoxicity illustrates the opposite pharmacovigilance problem, namely rare but clinically significant idiosyncratic injury. Flucloxacillin remains a well-characterised cause of cholestatic liver injury supported by large nationwide data, while co-amoxiclav is one of the most commonly implicated antibiotics in drug-induced liver injury registries [21,22]. These events are difficult to predict prospectively at the individual level, which makes them attractive targets for AI-enabled signal detection in large post-marketing datasets, though the translation from signal to clinical intervention remains limited.

### 3.2. Artificial Intelligence Approaches in Antibiotic Safety Monitoring

**Figure 1:**
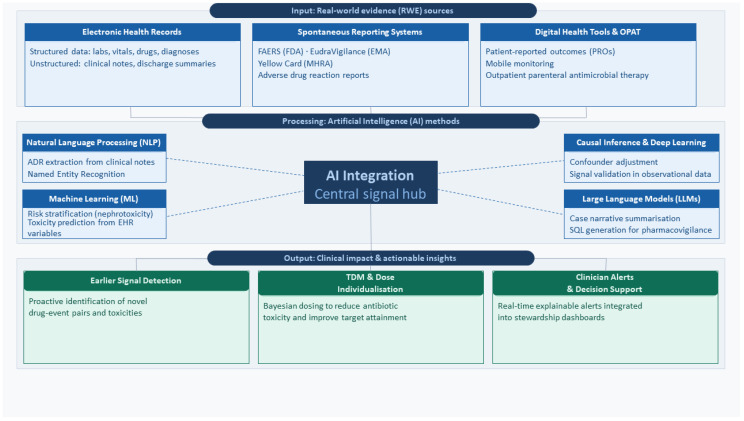
AI-enabled antibiotic safety monitoring ecosystem.

Figure 1 provides an overview of the AI-enabled antibiotic safety ecosystem. It is important to note at the outset that the contemporary AI literature in pharmacovigilance encompasses fundamentally different types of methods that are not interchangeable: classical machine learning (logistic regression, random forests, gradient boosting), deep learning (hierarchical neural networks, transformer architectures), natural language processing (NLP, including both rule-based and neural approaches), and generative large language models (LLMs). Each operates differently, has different evidence bases, and carries different implementation considerations. Conflating them as ‘AI’ without distinction obscures important methodological differences [23].

Real-world evidence is drawn from three principal input sources: electronic health records (structured laboratory, vital sign, medication, and diagnostic data, together with unstructured clinical notes and discharge summaries); spontaneous reporting systems (FAERS, EudraVigilance, and the Yellow Card scheme); and digital health tools including patient-reported outcomes and outpatient parenteral antimicrobial therapy (OPAT) platforms. These data streams are processed through a central AI integration hub employing four complementary methodological approaches: natural language processing for adverse drug reaction extraction and named entity recognition from clinical text; machine learning for nephrotoxicity risk stratification and toxicity prediction from structured variables; causal inference and deep learning for confounder adjustment and signal validation in observational data; and large language models for case narrative summarisation and pharmacovigilance query generation. Clinical outputs comprise earlier detection of novel drug-event pairs and toxicities, therapeutic drug monitoring and Bayesian dose individualisation to reduce antibiotic toxicity and improve target attainment, and real-time explainable clinician alerts integrated into antimicrobial stewardship dashboards.

FAERS, FDA Adverse Event Reporting System; MHRA, Medicines and Healthcare products Regulatory Agency; NLP, natural language processing; OPAT, outpatient parenteral antimicrobial therapy.

Machine learning models for individual toxicity prediction generally use structured variables such as renal function, age, cumulative drug dose, concomitant nephrotoxins, laboratory trends, and prior exposure history. Models of this type have been developed for vancomycin-associated nephrotoxicity, colistin nephrotoxicity, and linezolid thrombocytopenia, and these systems often demonstrate higher AUROC than simple threshold-based rules because they incorporate multiple interacting variables [24,25,26]. However, AUROC is insufficient as the sole performance metric: calibration (the agreement between predicted probabilities and observed outcomes), decision-curve analysis, and net reclassification must also be reported if models are to be assessed for clinical utility. Many published antibiotic AI models inadequately report calibration, limiting the ability to judge their real-world usefulness.

A more fundamental limitation is the predominance of single-centre, internally validated models. Local EHR structure, formulary composition, dosing conventions, laboratory reporting standards, and documentation culture all vary significantly between institutions. When antibiotic AI models developed in one hospital are applied externally, their performance frequently deteriorates. This problem of limited transportability is not unique to antibiotic safety, but it is particularly consequential in stewardship settings where pathways span emergency departments, wards, intensive care units, OPAT, and readmission encounters. External validation across multiple centres, ideally using common data model architectures, should be a minimum requirement before clinical adoption.

Natural language processing is valuable because many antibiotic ADRs are first documented in clinical free text before they are coded in structured fields. Clinicians describe confusion on cefepime, tingling on linezolid, or rash after beta-lactams in narrative notes, discharge summaries, and incident reports well before diagnostic codes are updated. Scoping reviews of supervised NLP for adverse drug event detection demonstrate that transformer-based architectures and ensemble systems can achieve strong performance on benchmark datasets, and that NLP may meaningfully augment the capacity to detect antibiotic-related safety signals in routine documentation [24,27,28,29]. However, performance on curated benchmarks does not guarantee generalisability to local EHR text, which typically differs in structure, completeness, and clinical vocabulary.

Deep learning extends NLP capability by learning complex representational features that are difficult to specify manually. Hierarchical recurrent networks and causal deep learning approaches have been applied to adverse drug event extraction from EHRs, and causal methods are especially relevant to antibiotics because nephrotoxicity and neurotoxicity frequently occur in patients whose baseline risk is already elevated due to severity of illness [25,26,28,29]. A model that does not account for confounding by indication, severity, and competing nephrotoxins will produce predictions that are neither clinically credible nor actionable. Addressing confounding is not a technical refinement; it is a prerequisite for clinical usefulness.

Bayesian approaches retain a distinct and important role in antibiotic safety monitoring. At the population level, Bayesian neural network methods for signal generation from spontaneous reporting data remain landmarks of pharmacovigilance methodology [30]. At the patient level, Bayesian logic underpins modern TDM and model-informed precision dosing, where measured drug concentrations are used to update prior pharmacokinetic estimates in order to individualise future dosing. Hybrid systems combining disproportionality methods, machine learning, and expert-defined Bayesian networks are increasingly used to prioritise signals or support causality assessment in pharmacovigilance [30,31,32,33]. For antibiotic stewardship, where clinicians already balance prior probability, measured concentrations, organ function, and time-course, Bayesian methods are conceptually well aligned with bedside reasoning.

### 3.3. Real-World Evidence Sources Used in AI-Based Safety Monitoring

AI-based antibiotic safety monitoring is constrained by the quality and completeness of the RWE sources on which models are trained and evaluated. EHRs are the most information-rich source because they combine medication administration records with laboratory trends, microbiology, physiological observations, clinical notes, and outcomes. This richness makes them well suited to exposure-linked toxicities such as vancomycin AKI, linezolid cytopenia, and cefepime neurotoxicity. Common data model approaches, including Observational Medical Outcomes Partnership (OMOP) implementations, improve portability by standardising coding across institutions, and recent work on pretrained patient trajectories suggests that multi-centre adverse drug event prediction is becoming technically more feasible [8,34]. However, EHR data quality problems, including poor timestamp fidelity, missing infusion details, unstable creatinine baselines, and inconsistent documentation of treatment cessation reasons, remain significant obstacles that are not resolved by applying more complex AI methods.

Spontaneous reporting systems remain central for population-level signal detection. The FDA Adverse Event Reporting System (FAERS), EudraVigilance within the European regulatory network, and the Yellow Card scheme of the MHRA each provide broad post-marketing visibility across products and jurisdictions. Their strengths include scale, sensitivity to unusual adverse event patterns, and the ability to detect rare or delayed toxicities that would not be apparent in single-centre studies. Their limitations are equally familiar: under-reporting, duplicate or incomplete submissions, uncertain causality, the absence of exposure denominators, and substantial heterogeneity in reporter expertise [4,5]. These limitations do not negate the value of spontaneous reporting systems; rather, they explain why AI methods are increasingly layered onto these data sources to support signal prioritisation, deduplication, and stratified review. FAERS-based disproportionality analyses have characterised safety signals for antistaphylococcal penicillins, while EudraVigilance analyses have explored persistent sensory adverse reactions associated with antibiotics [35,36]. These analyses generate hypotheses, not causal conclusions.

Administrative data and claims databases contribute a different dimension of RWE. They are typically less granular than EHRs for laboratory-defined toxicities, but useful for delayed outcomes, healthcare utilisation, treatment discontinuation, and multi-institutional comparative safety analyses. Cross-source triangulation, combining EHR data for clinical detail with administrative data for population coverage and spontaneous reports for signal sensitivity, represents the analytical framework most likely to support credible AI-enabled pharmacovigilance. This principle is consistent with regulatory expectations in RWE science and is directly applicable to antimicrobial stewardship contexts [8,9].

Wearables and remote monitoring represent the least mature RWE source in antimicrobial safety, but their relevance is growing, particularly for OPAT. Tele-OPAT studies indicate that remote contact can support safety surveillance through structured nurse-led review, adverse event prompting, and laboratory result triage [37,38,39,40]. For high-risk antibiotics, the most plausible near-term applications include remote monitoring of fever, heart rate, symptom burden, infusion adherence, and patient-reported warning signs, combined with scheduled laboratory surveillance. Evidence for antibiotic-specific wearable algorithms is currently very limited, and their clinical utility in this context has not been prospectively evaluated.

### 3.4. AI in Therapeutic Drug Monitoring for High-Risk Antibiotics

AI is most clinically established in one domain of antibiotic safety monitoring: TDM and dose individualisation. This is the area where the available evidence is strongest, the clinical benefit most credible, and the implementation most advanced. Vancomycin is the best-developed example. The revised consensus guideline endorsing AUC-guided dosing reflects the consistent association between excessive cumulative exposure and nephrotoxicity, and the inadequacy of trough-only monitoring as a surrogate [10]. Bayesian forecasting platforms use population pharmacokinetic models and sparse patient-level concentration measurements to estimate individual drug exposure, and comparative evaluations demonstrate substantial variation in model performance across patient populations and clinical settings, underscoring the importance of local validation rather than uncritical software adoption [41].

Prospective and implementation studies suggest that model-informed precision dosing may improve vancomycin target attainment and possibly improve clinical outcomes, though the evidence for the latter is less consistent. Validation studies in intensive care, paediatrics, and general hospital practice have shown that Bayesian tools are feasible in routine care when supported by pharmacists and standardised sampling workflows [42,43,44,45,46]. Critically, these tools shift the temporal window of safety intervention: rather than waiting for an overt serum creatinine rise, they allow clinicians to identify patients at risk of nephrotoxic exposure and adjust therapy earlier. This is the operationally important interface between AI-enabled TDM and stewardship: not replacement of clinical judgement, but earlier recognition and prevention of avoidable toxicity.

Aminoglycosides represent the historical foundation of Bayesian dosing in anti-infective pharmacology. The core logic, using measured concentrations and patient-specific parameters to update pharmacokinetic estimates and guide subsequent dosing, remains highly relevant in a modern model-informed precision dosing framework [12,47,48,49]. Beta-lactam TDM, although less consistently implemented, shows promise for improving target attainment in critically ill patients and for toxicity avoidance with high-exposure agents such as cefepime [50,51]. For linezolid, emerging pharmacometric and meta-analytic evidence suggests that exposure-guided dosing may reduce the incidence of thrombocytopenia, a clinically important finding given the prolonged treatment courses required in some indications [52].

**Figure 2:**
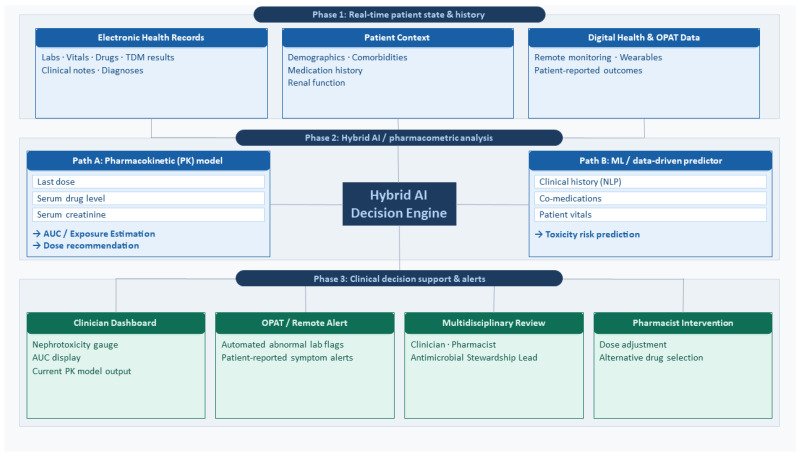
Hybrid AI decision engine for individualised antibiotic safety monitoring.

Machine learning adds an additional layer to TDM by complementing pharmacometric models with flexible prediction of toxicity risk from complex, interacting clinical variables. Recent studies have used machine learning to predict vancomycin-associated nephrotoxicity from AUC and clinical covariates, and to predict colistin nephrotoxicity from structured EHR data [53,54]. The key interpretive point is that machine learning is unlikely to replace Bayesian TDM, which rests on well-validated pharmacokinetic theory. A more realistic future is a hybrid architecture in which pharmacokinetic models estimate drug exposure while machine learning translates that exposure into dynamic, patient-specific toxicity risk estimates under real-world clinical conditions. Table 1 summarises major AI-enabled safety monitoring approaches across antibiotic classes. A representative clinical workflow integrating these components is presented in Figure 2.

The workflow operates across three sequential phases. Phase 1 integrates real-time patient state and clinical history from electronic health records (laboratory results, vital signs, drug administration records, therapeutic drug monitoring results, and clinical notes), patient context data (demographics, comorbidities, medication history, and renal function), and digital health and OPAT data (remote monitoring, wearables, and patient-reported outcomes). Phase 2 applies a hybrid AI and pharmacometric analysis through two parallel computational paths: Path A uses a pharmacokinetic model incorporating last dose, serum drug level, and serum creatinine to generate AUC and drug exposure estimates and derive dose recommendations; Path B applies a machine learning and data-driven predictor drawing on clinical history processed by natural language processing, co-medications, and patient vital signs to generate toxicity risk predictions. Both paths converge on a Hybrid AI Decision Engine. Phase 3 translates analytical outputs into four clinical decision support and alert channels: a clinician dashboard displaying nephrotoxicity gauge, AUC, and current pharmacokinetic model output; OPAT and remote alerts flagging abnormal laboratory values and patient-reported symptom changes; multidisciplinary review involving the responsible clinician, pharmacist, and antimicrobial stewardship lead; and pharmacist-led intervention encompassing dose adjustment and alternative drug selection.

AUC, area under the concentration-time curve; NLP, natural language processing; OPAT, outpatient parenteral antimicrobial therapy; PK, pharmacokinetic.

## 4. Discussion

### 4.1. Clinical Utility Versus Predictive Performance

A fundamental distinction that is frequently obscured in the AI pharmacovigilance literature is the difference between predictive performance and clinical utility. A model may demonstrate an AUROC of 0.85 and still contribute nothing meaningful to patient care if it generates excessive false-positive alerts, is not explainable to clinicians, cannot be integrated into existing workflows, or predicts outcomes too late for preventive action. The available evidence for antibiotic AI safety monitoring must be interpreted with this distinction in mind. The majority of published models have been evaluated solely on discrimination metrics, without calibration data, decision-curve analysis, alert fatigue assessment, or prospective impact studies.

Alert fatigue is a particularly important concern in the stewardship context. Antibiotic prescribers and pharmacists already manage high volumes of clinical decision support alerts, many of which are overridden without meaningful engagement. An AI model that generates numerous low-specificity toxicity warnings will not improve safety; it will reduce clinician engagement with all safety alerts. The design of AI-enabled antibiotic monitoring tools should explicitly target high positive predictive value in defined clinical populations, with clear thresholds for when alerts are generated and how they are communicated. Explainability, the capacity to identify which clinical variables are driving the prediction, is not optional in this context: it is necessary both for clinical trust and for the detection of model errors or biases.

Workflow integration is closely related to explainability. AI tools that require clinicians to consult a separate platform, re-enter data, or interpret opaque scores are unlikely to achieve adoption. The most successful implementations of clinical decision support in antimicrobial stewardship have been those embedded directly into EHR workflows, TDM consult services, or pharmacist-led review processes. Antibiotic AI safety monitoring tools should be designed with the same integration principle: they should deliver actionable information to the right clinician at the right clinical decision point, rather than generating background scores that require additional effort to interpret and act upon.

### 4.2. Implementation Barriers and Opportunities

The principal barrier to clinical implementation is not algorithm performance but data quality. Antibiotic toxicity is time-dependent and context-dependent. Serum creatinine trajectories are more informative than single values; platelet trends matter more than isolated counts; and narrative notes frequently document toxicity precursors before coded diagnoses are entered. Systematic reviews of EHR-based adverse drug event prediction demonstrate wide heterogeneity in feature engineering, outcome definitions, validation strategies, and reported performance metrics, which complicates translation into routine practice [55]. For antibiotic safety specifically, poor timestamp fidelity, missing infusion administration records, unstable creatinine baselines, and inconsistent documentation of treatment discontinuation reasons are recurring obstacles that AI methods alone cannot overcome.

Interoperability represents a closely related challenge. Models trained in a single hospital frequently fail when deployed elsewhere because formulary composition, laboratory reporting standards, dosing conventions, and documentation culture differ substantially between institutions. Common data model initiatives partially address this, and the emergence of multi-centre EHR learning systems and federated learning architectures is an encouraging development [8,34]. Even so, external validation remains the exception rather than the rule in published antibiotic AI studies, and there is no systematic benchmarking framework that would allow performance comparisons across models, institutions, or patient populations. Clinical pharmacologists and stewardship teams should be explicitly sceptical of models supported only by internal validation, particularly when their intended deployment setting differs from the development setting.

The regulatory landscape is evolving in directions relevant to implementation. Recent FDA guidance proposes a risk-based credibility framework for AI models used in drug and biologics safety monitoring, emphasising context of use, data provenance, validation requirements, and lifecycle governance. The European Medicines Agency reflection paper on AI throughout the medicinal product lifecycle similarly emphasises human oversight, bias control, data governance, documentation, and fitness for purpose. The EU AI Act introduces additional risk-stratification requirements for AI systems used in high-risk settings including healthcare. For antibiotic safety monitoring tools deployed in care delivery, the practical message is consistent: these tools should be governed as safety-critical software from the point of development rather than treated as general analytics utilities whose governance can be deferred.

Interpretability and explainability are therefore integral requirements, not technical refinements. Clinicians are unlikely to modify necessary anti-infective therapy on the basis of an opaque algorithmic score, particularly when the indication is serious, such as prosthetic joint infection, endocarditis, or persistent bacteraemia. Explainable AI methods applied to pharmacovigilance have shown that feature attribution approaches can identify which clinical variables most strongly drive adverse outcome predictions, supporting assessment of clinical plausibility [56]. In antibiotic practice, a model that identifies renal trajectory, cumulative drug exposure, vasopressor requirement, and co-administration of nephrotoxins as the primary predictors of nephrotoxicity risk is clinically intelligible and actionable. One that does not offer this interpretability will not achieve sustained clinical adoption.

Against these barriers, meaningful opportunities exist, particularly in existing stewardship infrastructure. Clinical pharmacologists, infectious diseases physicians, stewardship pharmacists, and nursing staff already function as human oversight layers in routine care: they reconcile drug exposure data with microbiology results, organ function trends, and treatment goals. AI tools designed to support rather than replace this process, by making safety surveillance more systematic, earlier, and more patient-specific, can be embedded into structures that already exist. Vancomycin TDM rounds, AMS authorisation workflows, OPAT laboratory review clinics, and multidisciplinary stewardship meetings are natural integration points because they already involve structured review and accountable decision-making [39,40,46,49].

OPAT settings deserve particular emphasis. High-risk antibiotic toxicity frequently emerges after hospital discharge, when clinical observation is sparser and attribution is delayed by days or weeks. Prospective OPAT studies document that adverse drug events are common, often occur within the first weeks after discharge, and are especially frequent with vancomycin and daptomycin [37]. Tele-OPAT and remote laboratory surveillance improve safety visibility, but antibiotic safety monitoring in OPAT continues to depend heavily on fragmented, manually coordinated workflows [38,39,40]. This environment is well suited to carefully governed AI support, particularly for prioritising abnormal laboratory results, identifying patients at highest near-term risk, and supporting dose adjustment decisions in collaboration with stewardship and clinical pharmacology services.

### 4.3. Large Language Models in Antibiotic Safety Monitoring: Opportunities and Limitations

Large language models (LLMs) have attracted substantial interest in antibiotic pharmacovigilance because of their capacity to process, summarise, and generate natural language, but their role in antibiotic safety monitoring specifically must be defined carefully, and current enthusiasm should be tempered by a clear-eyed assessment of their limitations.

It is essential to distinguish LLMs from the other AI methods discussed above. Classical machine learning and deep learning models are trained on structured or high-dimensional clinical data to predict defined outcomes, and supervised NLP models extract and classify information from labelled clinical text; generative LLMs, by contrast, are trained on vast general text corpora to predict plausible language rather than on antibiotic-specific clinical outcomes or pharmacological data, and their output does not carry the same statistical grounding as a trained predictive model. This distinction has direct implications for how LLMs should be validated, deployed, and governed in antibiotic safety monitoring.

The risks of LLMs in antibiotic safety monitoring are substantial and not yet resolved by available safeguards. Hallucination, the generation of plausible-sounding but factually incorrect output, is a documented and incompletely mitigated behaviour, and LLMs have been shown to fabricate drug interactions, invent references, and misattribute adverse event frequencies. Prompt instability limits reproducibility, hidden bias in training corpora may introduce systematic errors that are not apparent from surface outputs, and automation bias, the tendency to defer uncritically to authoritative-sounding output, is a particular concern for time-pressured antibiotic prescribing decisions. Submitting patient-identifiable information to externally hosted LLM services raises privacy and data leakage risks, medico-legal accountability for LLM-generated recommendations remains unresolved, and regulatory frameworks for LLMs as clinical decision support tools are currently underdeveloped.

The most credible near-term role of LLMs in antibiotic safety monitoring is administrative and cognitive workflow acceleration under expert supervision, not autonomous toxicity adjudication. High-confidence use cases include summarising case narratives from spontaneous reports, generating structured database queries for pharmacovigilance evidence pipelines, triaging incoming adverse event reports by seriousness, and assisting first-pass extraction of medication-event relationships from clinical notes subject to expert verification. Early evidence suggests LLMs can assist with SQL generation for pharmacovigilance workflows and may detect drug–drug interactions in narrative case reports at levels comparable to pharmacist review, though these findings require replication and prospective validation in antibiotic-specific settings [57,58,59,60]. LLMs should not be deployed for unsupervised causality assessment, autonomous safety signal confirmation, or generation of clinical recommendations without robust human oversight.

### 4.4. Future Research Priorities

The most important advances in AI-enabled antibiotic safety monitoring will require systemic improvements in evidence quality, not incremental refinements to model architecture. A dedicated research agenda should address the following priorities.

Multicentre prospective validation. The majority of currently published antibiotic AI models have never been tested prospectively or in a setting other than their development institution. Prospective validation studies, particularly those measuring impact on patient outcomes rather than model discrimination alone, are the most important unmet need in this field.

Federated learning. Given the interoperability challenges and data governance constraints on centralised model training, federated learning architectures that allow hospitals to train shared models across distributed datasets without sharing patient-level data are a logical and timely direction for antibiotic safety AI. Antibiotic-specific federated applications remain sparse but are technically feasible using existing common data model infrastructures [8,34].

Harmonised outcome definitions. Heterogeneity in how nephrotoxicity, neurotoxicity, thrombocytopenia, and other antibiotic ADRs are defined across studies severely limits cross-study comparability and meta-analytic synthesis. Consensus outcome definitions for the most important antibiotic toxicities, developed in collaboration between clinical pharmacologists, stewardship specialists, and biostatisticians, are a prerequisite for meaningful evidence accumulation.

Implementation and pragmatic trials. Prospective implementation studies that measure the effect of AI-assisted monitoring on clinically meaningful outcomes, including time to toxicity recognition, rate of dose modification, length of stay, and patient-reported outcomes, are essential to determine whether these tools add value beyond existing stewardship practice.

Stewardship integration studies. Optimising the embedding of AI tools within existing stewardship workflows, TDM services, OPAT clinics, and multidisciplinary rounds requires dedicated implementation research that addresses human factors, alert design, workflow disruption, and governance.

Explainability frameworks. Development and validation of explainability methods appropriate to antibiotic safety prediction tasks, including feature attribution, contrastive explanation, and uncertainty quantification, will be necessary before these tools can be responsibly deployed in high-stakes clinical decisions.

Benchmarking standards. The absence of standardised benchmarking platforms for antibiotic AI models prevents fair comparison across studies and institutions. Reporting standards analogous to TRIPOD (Transparent Reporting of a multivariable prediction model for Individual Prognosis or Diagnosis) should be adopted and enforced by journals publishing in this domain.

## 5. Conclusions

High-risk antibiotics sit at the intersection of therapeutic necessity and preventable harm. A recent narrative review addressed AI for infection management broadly, while the present review focuses specifically on safety monitoring and pharmacovigilance. The available evidence suggests that AI may improve safety monitoring under specific conditions: when applied to clinically rich real-world data, when targeted at well-defined clinical problems such as nephrotoxicity prediction, adverse event extraction from clinical notes, Bayesian dose optimisation, or OPAT surveillance, and when embedded within existing stewardship and pharmacology workflows rather than deployed as standalone prediction systems.

The strongest near-term use cases are clinically supervised, explainable tools integrated into TDM services, stewardship dashboards, and OPAT review processes, not autonomous decision-makers. The evidence base is predominantly retrospective, largely internally validated, and insufficiently focused on patient outcomes rather than model performance metrics. Prospective implementation data are sparse, and it would be premature to conclude that AI currently improves antibiotic safety in routine practice across heterogeneous clinical settings.

For policy and practice, the implication is that hospitals should prioritise validation-ready, explainable AI linked to antibiotic TDM, laboratory surveillance, and AMS governance frameworks rather than adopting generic prediction tools whose performance in their specific setting is unknown. Regulatory frameworks for AI as safety-critical software are evolving rapidly, and antibiotic safety tools should be developed within those frameworks from the outset. Clinical pharmacologists, infectious diseases physicians, and stewardship pharmacists are well positioned to lead local implementation, validation, and governance because they already manage the clinical decisions that AI seeks to support.

The field now requires multicentre prospective studies, rigorous external validation, harmonised outcome definitions, and pragmatic implementation research across both inpatient and OPAT settings. If these conditions are met, AI may ultimately move antibiotic pharmacovigilance from delayed recognition of established toxicity towards anticipatory, patient-specific safety management. The transition from promise to proven clinical utility, however, requires the same rigour that is demanded of any other clinical intervention.

## Figures and Tables

**Table 1:** Representative AI-enabled safety monitoring strategies for high-risk antibiotics.

Antibiotic or Class	Key Toxicity Focus	Dominant RWE Source	AI Approach	Validation	Maturity	XAI	Evidence
Vancomycin	Nephrotoxicity, excessive exposure	EHRs, TDM platforms	Bayesian AUC-guided dosing, ML toxicity prediction [10,42,43,44,45,46]	Internal + some external validation	Moderate–high	Moderate (SHAP used in some models)	Prospective implementation studies exist [44,45]
Aminoglycosides	Nephrotoxicity, ototoxicity	TDM datasets, EHRs	Bayesian feedback dosing, model-informed precision dosing [12,47,48,49]	Mostly internal	Moderate	Low	Largely retrospective or observational
Colistin	Nephrotoxicity	EHRs, ICU datasets	ML using structured clinical variables [13,53]	Internal only	Early	Low	Retrospective only
Linezolid	Thrombocytopenia, neuropathy	EHRs, TDM datasets	Risk prediction models, personalised dosing [15,20,51,52]	Mostly internal	Early–moderate	Low	Retrospective; some PK modelling studies
Cefepime/beta-lactams	Neurotoxicity, excessive exposure	EHRs, ICU datasets	TDM-assisted exposure assessment, emerging ML [17,50,51]	Internal only	Early	Low	Retrospective
Antistaphylococcal penicillins, co-amoxiclav	Rare hepatotoxicity, sensory ADRs	FAERS, EudraVigilance, Yellow Card	Disproportionality, ML signal prioritisation [31,32,33,35,36]	Cross-system	Moderate (signal detection); low (bedside)	Low	Signal-level; no implementation trials
OPAT regimens (all classes)	Delayed ADRs, adherence, line complications	Remote monitoring, tele-OPAT, patient-reported data	Telemonitoring workflows; future wearable-assisted alerts [37,38,39,40,41]	Mostly single-centre	Early	Low	No prospective AI-specific implementation data

ADR, adverse drug reaction; AUC, area under the concentration-time curve; EHR, electronic health record; ICU, intensive care unit; ML, machine learning; OPAT, outpatient parenteral antimicrobial therapy; PK, pharmacokinetic; RWE, real-world evidence; SHAP, SHapley Additive exPlanations; TDM, therapeutic drug monitoring; XAI, explainable artificial intelligence.

## Data Availability

Data are available upon reasonable request from the corresponding author.
